# Arbuscular Mycorrhizal Fungi and Dry Raw Garlic Stalk Amendment Alleviate Continuous Monocropping Growth and Photosynthetic Declines in Eggplant by Bolstering Its Antioxidant System and Accumulation of Osmolytes and Secondary Metabolites

**DOI:** 10.3389/fpls.2022.849521

**Published:** 2022-03-31

**Authors:** Muhammad Imran Ghani, Ahmad Ali, Muhammad Jawaad Atif, Muhammad Ali, Bakht Amin, Zhihui Cheng

**Affiliations:** ^1^College of Horticulture, Northwest A&F University, Xianyang, China; ^2^Horticultural Research Institute, National Agricultural Research Centre, Islamabad, Pakistan

**Keywords:** arbuscular mycorrhizal fungi (AM fungi), antioxidant system, dry raw garlic stalk, continuous monocropping, eggplant, osmolytes, secondary metabolites

## Abstract

Vegetable production under plastic sheds severely threatens regional eco-sustainability *via* anthropogenic activities (excessive use of agrochemicals, pesticides) and problems associated with replanting. Long-term successive cropping across growing seasons induces continuous cropping stress, whose effects manifest as diminished plant growth. Therefore, it is imperative that we develop environmentally sustainable approaches, such as replacing agrochemicals with vegetable waste like dry raw garlic stalk (DRGS) or use biofertilizers like arbuscular mycorrhizal fungi (AMF) (e.g., *Diversispora epigaea*). In this study, the influence of AMF on the growth, biochemical attributes, antioxidant defense system, phytohormones, accumulation of osmolytes, phenols, and mineral elements in eggplant grown on DRGS-amended soils under continuous monocropping (CMC) was studied. The results showed that inoculation with AMF or the DRGS amendment could improve the pigments’ content, photosynthesis, and antioxidant defense system; augmented phytohormones synthesis (except for ABA), and increased the leaves’ mineral nutrients. These parameters were enhanced most by the combined application of AMF and DRGS, which also increased the concentration of osmolytes, including proline, sugars, and free amino acids in eggplant when compared with the control. Furthermore, either AMF and DRGS alone, or in combination, ameliorated the induced stress from continuous cropping by reducing the incidence of *Fusarium* wilt and production of ROS (reactive oxygen species); lipid peroxidation underwent maximal reduction in plants grown under the combined treatments. The AMF, DRGS, and AMF + DRGS exhibited a lower disease severity index (33.46, 36.42, and 43.01%), respectively, over control. Hence, inoculation with AMF coupled with DRGS amendment alters the photosynthetic attributes in eggplant through the upregulation of its antioxidant system and greater accumulation of osmolytes, which led to the improved growth and yield of eggplant.

## Introduction

Eggplant (*Solanum melongena*) is a commercially important cash crop that is often cultivated as a monoculture under plastic sheds in China (Wang et al., 2015). In general, crops under monocropping incur additional stresses, including fungal and pest infestations, diminished soil physicochemical characteristics, and gradual accumulation of root exudates in soil, which collectively impose consistent impediments to robust plant growth (Wang et al., 2015; Ali et al., 2019a, b, 2021a). These various biotic and abiotic stresses cause changes in the crop plant by disrupting its balance of reactive oxygen species (ROS). Overproduction of ROS impairs membrane integrity and cellular function by interfering with photosynthesis, mineral absorption, and assimilation (Naciri et al., 2021). To mitigate elevated ROS production and metabolic protection, plants will often upregulate their antioxidant system and accumulate more osmolytes (Ghani et al., 2019a; Ahanger et al., 2021). Antioxidants are enzymatic and non-enzymatic components found in almost every cellular organelle, where they help to stabilize cells by removing excess ROS; different kinds of osmolytes, including amine, compounds (glycine betaine), proline, and soluble sugars, are key regulators of water content, enzyme functions, and stress signaling (Ahanger et al., 2021). Therefore, bearing in mind these concerns, it is crucial to devise management techniques that augment these innate tolerance mechanisms for protecting the growth and production capacity of agricultural crops under different environmental conditions with a concomitant reduction in chemical fertilization and amelioration of obstacles associated with continuous cropping. The paramount strategies to do this include using beneficial microorganisms or incorporating crop residues, which, directly or indirectly, can improve the tolerance mechanisms of the target crop.

Arbuscular mycorrhizal fungi (AMF) are key members of the phylum *Glomeromycota* that exist in soil, where they are capable of symbiotically colonizing the roots of more than 85% of plant families, including many vegetables and horticultural plants (Emmanuel and Babalola, 2020). This mutualistic symbiotic relationship involves phytohormonal signaling between AMF and its host plants for interspecies communication (Wang et al., 2021). AMF modulates plant growth and development by forming a hyphal network with the host plant’s roots and enhancing its efficacy of mineral nutrient uptake (Rouphael et al., 2015), accelerating its growth, as well as modifying the biosynthesis of several metabolites, such as free amino acids, fatty acids, and phytohormones to address different stresses (Begum et al., 2020). AMF inoculation can ameliorate stress-induced damage to plants’ growth and biomass accumulation by minimizing the production of harmful radicals such as H_2_O_2_, thereby reducing membrane damage by promoting the activity of antioxidant enzymes (Gómez-Bellot et al., 2021). AMF may also induce the production of phytoalexins and antioxidant enzymes in plants, triggering the pathogen defense systems in host plants (Maharshi et al., 2019).

China is, currently, the world’s largest producer of garlic, contributing over 0.02 billion tons annually, which also entails abundant raw garlic stalks as a by-product, a significant cause of environmental pollution. Almost 70% of garlic stalk is discarded or burned by farmers because they consider it waste (Lee et al., 2017). To address this issue, incorporating this waste (i.e., DRGS) in soil would offer an effective way to utilize this resource as a plant fertilizer and improve crop yields. Furthermore, incorporating crop residues in soil has many benefits, such as increasing the soil nutrients available for crop production, increasing the water-holding capacity of soil, and inputting organic matter, which reduces soil-borne pathogens (Ghani et al., 2019a,b). In recent years, considerable attention has been paid to the allelopathic potential of garlic crops as a novel strategy for crop production, this offering the most stable and resilient environmental benefits in terms of simultaneously reducing chemical fertilizer, regulating crop production, improving plants’ physiology, soil biological alterations, and ensuring ecological sustainability (Ali et al., 2019a,c,d, 2020, 2021b; Ghani et al., 2019a,b; Ali M. et al., 2021). Therefore, incorporating DRGS in the soil alone or combined with AMF may offer a viable alternative due to its cost-effectiveness and environmentally friendly biological sources. This could provide a wide range of opportunities to alleviate CMC-related obstacles in intensive vegetable production systems and, perhaps, serve as a substitute for chemical fertilizer. Yet, very few scientific investigations to date have assessed both plant growth and resistance-associated benefits of soil amended with DRGS (dry raw garlic stalk). Therefore, we hypothesized that AMF inoculation and DRGS incorporation would be able to eliminate continuous cropping obstacles in eggplant by modulating its antioxidant dynamics, osmolytes’ metabolism, root activity, mineral absorption, and nutrient assimilation.

The objectives of this study were (1) co-application of DRGS and AMF would be able to enhance eggplant growth and yield, and the effect of dual treatment could be greater than a single treatment; and (2) the co-application of AMF and DRGS improved plant physiological and biochemical attributes more than a single application.

## Materials and Methods

### Experimental Site Description, Soil and Organic Material Collection, and Arbuscular Mycorrhizal Fungi Propagation

A 9-month-long pot experiment (March through November 2018) was conducted in the horticulture field of Northwest A&F University in Yangling, China (34°17′N, 108°4′E). The soil was collected from the plastic shed of Northwest A&F University (Yangling, China) where eggplant was continuously cultivated for 5 consecutive years. This soil is classified as anthrosol, according to the Food and Agriculture Organization (FAO). The basic soil and DRGS characteristics are given in Supplementary Table 1. The soil used in the pots is best described as replanted stressed soil; it is somewhat low in organic carbon input and suffering from soil-borne diseases, especially *Fusarium* wilt (Ghani et al., 2019a,b). The organic substrate, i.e., garlic stalk, was collected from the garlic field of Yangling, and the proposed organic material input consisted of garlic leaves and stalk. Briefly, the garlic leaves and stalks were harvested and air-dried, mechanically crushed, and homogenized into powder and stored in the dark at room temperature before their use. The AMF isolates of *Diversispora epigaea* [formerly known as *Glomus versiforme*; Sun et al. (2019)] were used in this experiment. Briefly, the AMF inoculum consisted of spores and infested maize roots. The inoculum was reproduced under controlled greenhouse conditions (i.e., 25°C/16°C day/night temperature with 70–75% relative humidity), as described in our previous experiment (Ali et al., 2019a).

### Plant Material and Treatment Set-Up

The eggplant (*S. melongena* L. cv. “Tai Kong Qie Wang”) seeds were surface sterilized before being sown in germination trays (35 cm × 21 cm) filled with a commercial seedling medium containing 20–25% organic matter, 8–10% humic acid, and pH 6.5–6.8 as previously described by Atif et al. (2019, 2020, 2021). These trays were moved into the growth chamber and kept there at 27°C. About 3-week-old uniform eggplant seedlings with true leaves were transplanted into pots (30 cm × 24 cm) containing 8 kg of soil. Before transplanting the seedlings in pots, the DRGS and soil were thoroughly mixed. DRGS was applied alone or in combination with AMF at a rate of 3 g/100 g of dry soil; this was selected as the best treatment based on our previous experiment (Ghani et al., 2019a,b). The DRGS pots without AMF inoculation were supplemented with 25 g of autoclaved inoculum. The inoculum was kept near the roots of eggplant seedlings by drenching. The experiment was conducted with four treatments replicated three times for 30 plants per treatment with a total of 120 plants used: (1) Control, CK: without DRGS and AMF; (2) AMF (without DRGS); (3) dry raw garlic stalk (DRGS) without inoculum; and (4) AMF + DRGS. Each pot was fertilized one time with organic fertilizer (PengDiXin “manure replacement,” Henan, China) at a rate of 10 g and 10 g of compound fertilizer (81:18:18 of N-P_2_O_5_-K_2_O), which was applied as basal fertilizer, in accordance with local guidelines. Throughout the experimental period, standard agronomic practices were followed to maintain the plants. Growth, physiological, and biochemical variables of eggplant were measured at four different developmental stages: 1st flowering stage, 1st fruiting stage, 2nd flowering stage, and 2nd fruiting stage.

### Measurement of Plant Growth, Photosynthetic Pigment, and Gas Exchange Parameters

Five plants were randomly selected from each replication, and 15 plants were assessed for each treatment to quantify morphological traits at different growth stages. Plant height was recorded using a measuring tape, and an electronic vernier caliper was used to measure a stem diameter.

To extract total chlorophyll contents (0.25 g), fresh leaves of eggplant were immersed in 80% acetone (V/V), kept solution at room temperature for 48 h, followed by centrifugation at 10,000 rpm for 5 min. Observance of chlorophyll a and chlorophyll b was detected using OD646 and OD663 following the procedure (Lichtenthaler and Wellburn, 1983).

Net photosynthetic rate (Pn), stomatal conductance (gs), intercellular CO_2_ concentration (Ci), and transpiration rate (E) were measured in the uppermost leaf by using a LI-6400 portable photosynthesis system (Li-Cor, Lincoln, NE, United States). In addition, a chlorophyll fluorometer (PAM-2000 chlorophyll fluorometer) was used to determine maximal photosystem II (PSII) (*F*_v_/*F*_m_, ΦPSII) after 30-min dark adaption. The recorded data were processed by PAM Win software.

### Estimation of Antioxidant Enzymes, Oxidative Stress Biomarkers, and Membrane Stability Index

Fresh tissue (0.5 g) was immersed in a chilled 0.05-mM (pH 7.8) phosphate buffer containing 0.1% polyvinylpyrrolidone and 0.5-m Ethylenedinitrilotetraacetic acid (EDTA) and grounded in a pre-chilled pestle and mortar. The reaction mixture was centrifuged at (12,000 × *g*) for 20 min at 4°C, and the supernatant was collected for enzyme analysis.

For the estimation of superoxide dismutase (SOD), we have followed the protocol of Dhindsa and Matowe (1981) by quantifying the photochemical degradation of NBT (nitro blue tetrazolium). The Peroxidase (POD) activity in eggplant leaves was assessed by the Guaiacol procedure (Bestwick et al., 1998). To evaluate Catalase (CAT) activity, we have adapted the proposed procedure of Chance and Maehly (1955). To evaluate ascorbate peroxidase (APX) in eggplant leaves, Nakano and Asada (1981) procedure was employed.

Polyphenol oxidase (PPO) activity was assayed by calculating the initial rise in absorbance at 410 nm within the first 3 min of the reaction (Zheng et al., 2007). Phenylalanine ammonia-lyase (PAL) activity was evaluated following the procedure of Gao (2006a). To quantify the glutathione (GSH) and ascorbic acid (AsA) content, we have employed the method of Moron et al. (1979) and Omay et al. (1979), respectively. For the calculations, the standard curve of AsA and GSH curves were employed.

The concentration of H_2_O_2_ and O_2_^–^ was quantified following the method of Zhang et al. (2010). For lipid peroxidation, the content of malonaldehyde (MDA) was measured by incubating tissue extract with thiobarbituric acid (TBA) at boiling temperature (Velikova et al., 2000).

The membrane stability index (MSI) was calculated using the method given by Shanahan et al. (1990) using the following formula MSI = [1−(C1/C2)] × 100.

### Estimation of Mineral Nutrients, Total Phenol, Osmolytes, and Protein Content

In order to measure N, P, and K, oven-dried tissue was digested in hydrochloric acid, and hydrogen peroxide was added until the solution became transparent. The Sabine (1962) technique for nitrogen determination was employed. The flame photometer was used to assess potassium in conjunction with continuous flow systems. Finally, the spectrophotometric method was used to determine phosphorus (Olsen, 1954).

The Singleton and Rossi (1965) method was used to estimate phenols. About 500 mg of dry sample was extracted in 80% ethanol, and then 0.1 ml of the supernatant was treated with Folin–Ciocalteu reagent, and the absorbance was measured at 765 nm. For the calculations, a gallic acid standard curve was employed.

The method employed for estimation of proline is described in detail by Bates et al. (1973). Soluble sugar was estimated according to the method described by Gao (2006b), and the free amino acid was measured following the method of Sadasivam (1996). For protein content assessment, we have followed the procedure of Bradford (1976) using BSA as a standard.

### Root Length, Root Weight, Arbuscular Mycorrhizal Fungi Colonization, and Root Activity

The root length was measured manually with a measuring tape. The plant and root fresh weight (FW) were determined immediately after harvesting, whereas the dry weight (DW) was determined after oven drying at 60°C for 48 h. The plant roots were placed in a formalin–acetic acid–alcohol (FAA) solution, cleaned, and cut into 2-cm long pieces before being preserved in 5 percent KOH for 30 min at 90°C. According to Phillips and Hayman (1970), the eggplant roots were then stained with trypan blue (0.05 percent). The tissues of dyed roots were examined using a microscope model (Olympus-Japan). To calculate the percentage of root colonization in eggplant roots, we have employed the method of McGonigle et al. (1990). Furthermore, 50 root pieces (0.5–1 cm) per treatment were used to analyze the root colonization percentage according to the formula given below:

Root colonization (%) = Number of root segments (colonized)/Number of root segments (observed) × 100

Root activity was quantified by using Triphenyl tetrazolium chloride (TTC). In brief, 0.2 g of eggplant roots was mixed with 5 ml of 4% TTC in 5-ml Na_2_HPO_4_ (Clemensson-Lindell, 1994). Activity was assessed by the formula reported as TTC-reducing intensity (mg g^–1^ h^1^).

TTC-reducing intensity = [TTC reduction mass/root weight × time].

### Endogenous Phytohormones’ Extraction, Purification, and Quantification

Approximately, 2 g of fresh eggplant leaves was weighed at different growth stages (i.e., 1st flowering, 1st fruiting, 2nd flowering, and 2nd fruiting stage), and fresh roots were weighed after harvesting. For the extraction and purification of indole acetic acid (IAA), abscisic acid (ABA), jasmonic acid (JA), and salicylic acid (SA), this was done following the method proposed by Pan et al. (2010). Approximately, 2 μL of d5-IAA (2 ng/μL), 25 μL of d6-ABA (0.25 ng/μL), 40 ng of D_4_-SA (ng/μL), and 2 μL of H_2_-JA (2 ng/μL) were added to each sample of eggplant leaves and roots as internal standards (Shanghai Yuanye Bio-Technology Co., Ltd., Shanghai, China). Next, 0.5 ml of an extraction solvent (isopropanol: H_2_O: concentrated HCl = 2:1:0.002, v/v/v) was added and shaken for 30 min at 4°C at a speed of 100 rpm. Then, 1 ml of dichloromethane was added, and the mixture was shaken for 30 min. The solvent was recovered after centrifugation and concentrated using a nitrogen evaporator. To measure the phytohormones’ content, HPLC-MS in a multiple-reaction monitoring (MRM) mode was used. An Agilent 1260 HPLC system equipped with an AB Qtrap 5500 triple quadrupole mass spectrometer with an electrospray ionization source was used. Each sample was injected into an Agilent SB-C18 column (50 mm × 4.6 mm, 1.8 m) and separated by the mobile phase at a flow rate of 0.8 ml/min as follows: acetonitrile (A) and distilled water with 0.1% acetic acid (B) for IAA, ABA, SA, and JA, and acetonitrile (A). The HPLC gradient program and multiple reaction monitoring (MRM) settings were utilized to measure each phytohormone. Injection volumes for IAA, ABA, SA, and JA were the same (5.0 L).

### Quantification of Fusarium Wilt Disease Severity Index and Eggplant Yield

The disease was monitored on a daily basis using a procedure previously suggested by Wang et al. (2016). The disease severity index was graded on a 0–4 scale (1:25% wilted leaves, 2:25–50% wilted leaves, 3:50–75% wilted leaves, and 4:75–100% wilted leaves). The following formula was used to calculate the disease index:


$$\begin{array}{c} Diseaseseverity\\ =\frac{\sum \mathrm{Disease}\mathrm{grade}\times \mathrm{Nnumber}\mathrm{of}\mathrm{plants}\mathrm{in}\mathrm{each}\mathrm{grade}}{\mathrm{Total}\mathrm{Nnumber}\mathrm{of}\mathrm{plants}\times \mathrm{highest}\mathrm{disease}\mathrm{grade}}\times 100 \end{array}$$

The eggplant yield (marketable size fruits) was collected weekly to calculate the eggplant yield.

### Statistical Analysis

Data were evaluated using two-way ANOVA as a 4- × -2 (treatment × sampling stage) factorial design for the trial using SPSS software v18.0. Differences in their means were determined by using Tukey’s *post hoc* test (deemed significant at *p* < 0.05). The Pearson correlation analysis was elucidated as a correlation matrix to illustrate the relationship between yield and different physiological and biochemical parameters of eggplant.

## Results

### Effect of Arbuscular Mycorrhizal Fungi and Dry Raw Garlic Stalk on Eggplant’s Growth and Its Root Morphology and Anatomy

**FIGURE 1:**
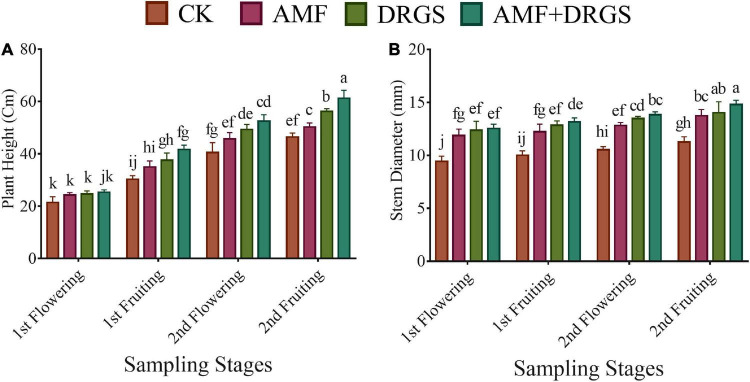
Effect of AMF inoculation and DRGS treatment on **(A)** plant height and **(B)** the stem diameter of eggplant. Data represent the means of ±S.E. (*n* = 5). Different letters show significant differences within treatments. Tukey’s HSD test was employed to verify statistical significance at *p* < 0.05.

**FIGURE 2:**
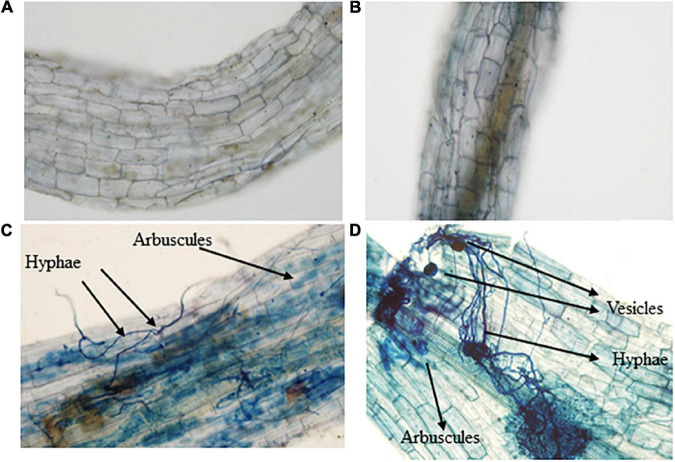
Microscopic pictures of eggplant roots and Arbuscular mycorrhizal fungal colonization. **(A)** Control plants’ roots, **(B)** dry raw garlic stalk roots, **(C)** arbuscular mycorrhizal fungal-inoculated plant roots, and **(D)** combination of Arbuscular mycorrhizal fungal and dry raw garlic stalk.

Application of AMF and DRGS alone or in combination significantly improved the plant growth variables. Plant height and a stem diameter increased at all growth stages. Compared with control, under AMF + DRGS, both plant height and stem-diameter increment increased maximally, respectively, by 31.84 and 31.34%, at the 2nd fruiting stage, followed by DRGS and AMF (Figures 1A,B). Inoculation with AMF (*D. epigaea*) and DRGS promoted root colonization in comparison with eggplant cultivated in CMC soil (Table 1). AMF effectively colonized the eggplant roots grown in CMC soil, and the colonization rate under the sole application of AMF was 37%, which was increased 52% under the joint DRGS and AMF amendment (Table 1 and Figures 2A–D).

**TABLE 1:** The effect of AMF inoculation and DRGS application on shoot fresh and weight, root length, root fresh and dry weight, yield, root AMF colonization%, and disease severity index.

Treatment	Shoot fresh weight (g pot^–1^)	Shoot dry weight (g pot^–1^)	Root length (cm)	Root fresh weight (g pot^–1^)	Root dry weight (g pot^–1^)	Root activity (mg^–1^ g^–1^ h)	Yield (g pot^–1^)	Colonization%	Disease severity index (%)
CK	305.09 ± 3.52d	73.30 ± 0.38d	21.50 ± 1.02d	44.17 ± 1.89d	12.20 ± 1.00d	13.30 ± 1.18d	2500 ± 5.15d	–	53.86a
AMF	333.21 ± 2.85c	84.09 ± 1.53bc	26.67 ± 1.89c	49.53 ± 1.28c	16.04 ± 0.47c	15.50 ± 1.10c	3300 ± 4.25c	37.00 ± 1.13b	35.84b
GS	389.66 ± 2.64b	89.82 ± 1.64b	29.17 ± 0.76b	67.57 ± 0.50b	18.26 ± 1.08b	18.21 ± 0.71b	3700 ± 6.13b	–	34.24c
AMF + GS	423.84 ± 1.16a	103.4 ± 1.34a	33.50 ± 1.50a	78.33 ± 2.93a	21.69 ± 1.36	20.70 ± 1.70a	4500 ± 5.32a	52.66 ± 1.69a	30.69d

*Data represent the mean of ±SE (n = 5). Different letters show significant differences within treatments. Tukey’s HSD test was employed to verify statistical significance at p < 0.05.*

The impacts of AMF and DRGS alone or in combination on the root length, root fresh weight, and root dry weight are presented in Table 1. Treatment with AMF, DRGS, and AMF + DRGS significantly increased root length, root fresh weight, and root dry weight, with the highest increment occurring in response to the AMF + DRGS application, being 59.52, 77.35, and 64.09% greater than the control, respectively. Relative to the control, AMF and DRGS increased root length by 17.14 and 38.88%, respectively, while root fresh weight was increased by 12.15% in AMF-treated plants and by 52.15% in DRGS-treated plants. A similar pattern was evident for root dry weight (Table 1). Similarly, root activity was also higher in all treatments *vis-à-vis* the control. Under AMF + DRGS, DRGS, and AMF, root activity was increased over the control by 55.60, 39.96, and 16.51%, respectively (Table 1).

### Effect of Arbuscular Mycorrhizal Fungi and Dry Raw Garlic Stalk on Chlorophyll Pigments, Photosynthesis, and the Photosystem II System

**FIGURE 3:**
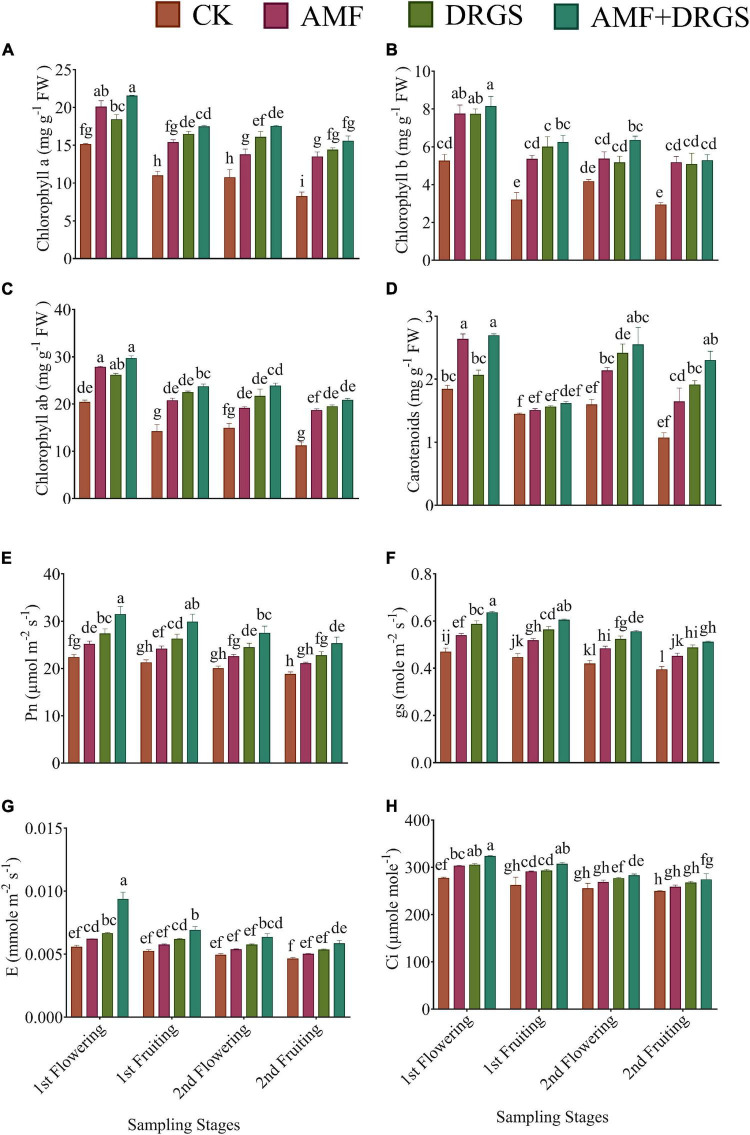
Effect of AMF inoculation and amendment with dry raw garlic stalk on **(A)** chlorophyll *a*, **(B)** chlorophyll *b*, **(C)** chlorophyll *ab* and **(D)** carotenoids content, **(E)** photosynthesis (Pn), **(F)** stomatal conductance (gs), **(G)** transpiration rate (E), and **(H)** internal CO_2_ (Ci) on eggplant leaves at 1st flowering, 1st fruiting, 2nd flowering, and 2nd fruiting stages. Data represent the means of ±S.E. (*n* = 5). Different letters show significant differences within treatments. Tukey’s HSD test was employed to verify statistical significance at *p* < 0.05.

Relative to the control, the application of AMF and DRGS alone or in combination enhanced the synthesis of chlorophyll *a*, chlorophyll *b*, chlorophyll *ab*, and carotenoid content at all growth stages (Figures 3A–D). Maximum chlorophyll *a*, chlorophyll *b*, and chlorophyll *ab* were observed in the 1st flowering stage. Relative to the control, chlorophyll *a* (37.99%), chlorophyll *b* (55.01%), and chlorophyll *ab* (42.96%) increased significantly under AMF + DRGS in the 1st flowering stage. In AMF-treated plants, the percentage increases in chlorophyll *a*, chlorophyll *b*, and chlorophyll *ab* were 32.80, 47.42, and 36.57%, respectively, over the control; for DRGS-treated plants, the corresponding increases were 32.80, 47.55, and 28.35%, but with no significant difference observed for chlorophyll *b* in the 1st flowering stage. Similarly, carotenoid content was higher in AMF- and DRGS-treated plants than the control (Figure 3D).

Inoculation with AMF and the DRGS amendment significantly improved photosynthesis and also ameliorated the negative effects of continuous cropping (Figures 3E–H). The Pn, gs, E, and (Ci) were higher in all treatments compared with control. Overall, Pn, GS, E, and Ci were higher in the 1st flowering and 1st fruiting stages than later stages (2nd flowering, 2nd fruiting). The AMF + DRGS treatment resulted in Pn increasing by 40.62, 39.68, 37.63, and 34.70% over the control in the 1st flowering, 1st fruiting, 2nd flowering, and 2nd fruiting, respectively (Figure 3E). Furthermore, AMF, DRGS, and AMF + DRGS enhanced gs (Figure 3F). Under the AMF + DRGS treatment, in the 1st flowering stage, a maximum increase of 35.40% in gs was observed compared with the control. In addition, the transpiration rate increased by 16.72% in the AMF + DRGS treatment compared with the control (Figure 3G). Similarly, the Ci was maximized in the 2nd fruiting stage, when it was 10.02% higher than the control (Figure 3H).

**FIGURE 4:**
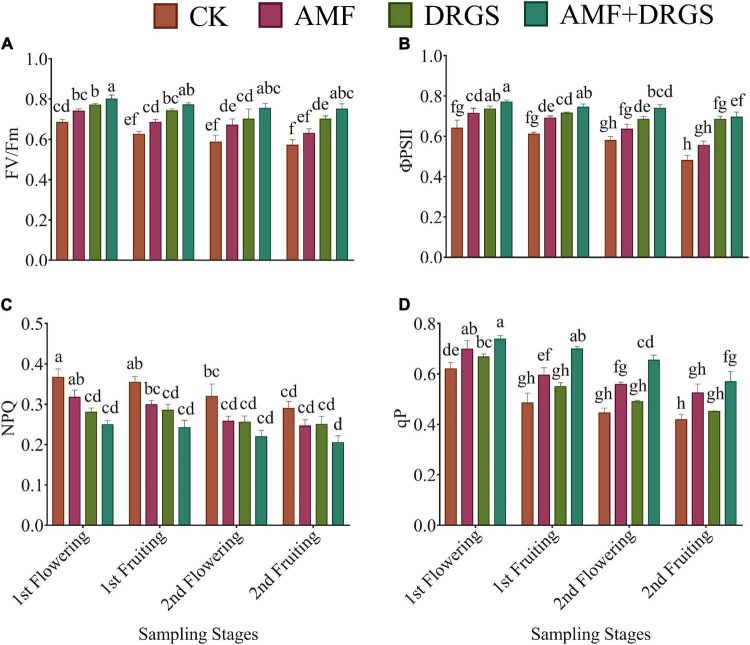
Effect of AMF inoculation and DRGS on maximal **(A)** photochemical efficiency (*F*_*v*_/*F*_*m*_), **(B)** chlorophyll photosystem II (ΦPSII), **(C)** non-photochemical quenching coefficient (NPQ), **(D)** photochemical quenching (qP) on eggplant leaves at 1st flowering, 1st fruiting, 2nd flowering, and 2nd fruiting stages. Data represent the means of ±S.E. (*n* = 5). Different letters show significant differences within treatments. Tukey’s HSD test was employed to verify statistical significance at *p* < 0.05.

Applying AMF and DRGS enhanced the PSII activity (*F*_V_/*F*_M_) in comparison with the control (Figures 4A–D). The FV/FM was greatest in the 1st flowering and 1st fruiting stages compared with later stages. FV/FM was significantly higher at the 1st flowering stage under the AMF + DRGS, DRGS, and AMF treatments by 16.88, 12.86, and 8.12% over the control, respectively (Figures 4B,D). A similar trend was observed in the 1st fruiting stage, 2nd flowering stage, and 2nd fruiting stage. Inoculation with AMF and incorporation of DRGS significantly stimulated ΦPSII in DRGS-, AMF-, and AMF + DRGS-treated plants compared with the control. Overall, a maximum ΦPSII was observed in 1st flowering stage vis-à-vis the other stages. AMF induced an increase of 11.32%; DRGS, 15.03%, and AMF + DRGS increased by 20.10% compared with control. Similarly, qP showed a similar trend, in that it was highest in the 1st flowering stage compared with the other stages and augmented most in AMF + DRGS-treated plants compared with the control. However, NPQ was reduced in all treatments relative to the control, with the maximum reduction occurring in the 2nd fruiting stage, being 15.07, 13.57, and 29.24% lower in AMF, DRGS, and AMF + DRGS than the control, respectively (Figure 4C).

### Effect of Arbuscular Mycorrhizal Fungi and Dry Raw Garlic Stalk on Osmolytes, Total Phenolic, Protein Content, and Phenylalanine Ammonia-Lyase Activity

**FIGURE 5:**
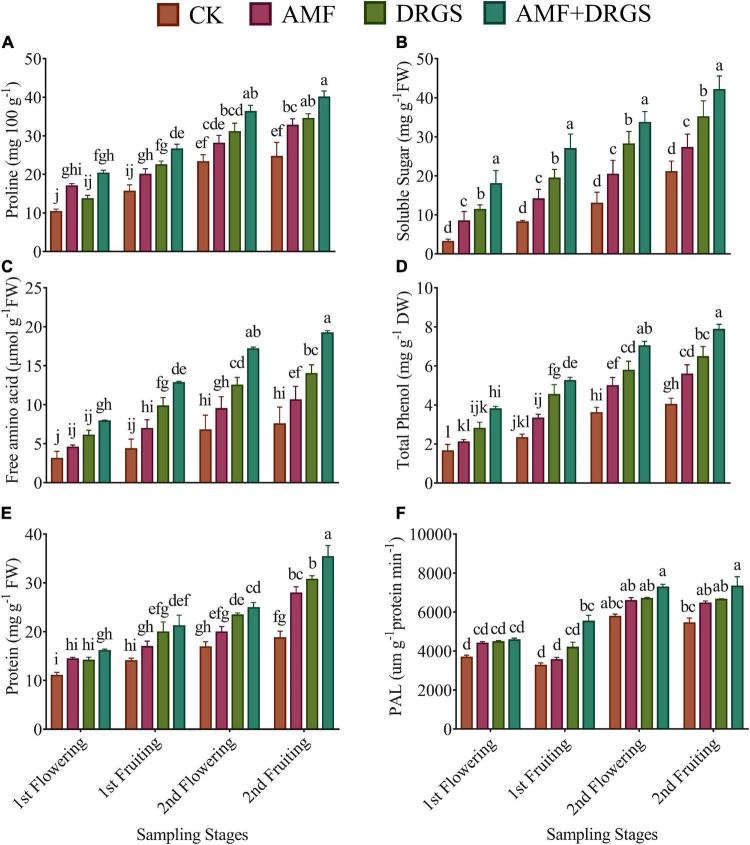
Effect of AMF inoculation and amendment with dry raw garlic stalk on proline **(A)**, soluble sugar **(B)** and amino acid **(C)** total phenol **(D)**, protein **(E)**, and Phenylalanine ammonia-lyase (PAL) **(F)** on eggplant leaves at 1st flowering, 1st fruiting, 2nd flowering, and 2nd fruiting stages. Data represent the means of ±S.E. (*n* = 5). Different letters show significant differences within treatments. Tukey’s HSD test was employed to verify statistical significance at *p* < 0.05.

The AMF and DRGS treatments enhanced the proline, soluble sugar, and amino acid levels (Figures 5A–C) at all growth stages compared with the control. Overall, a trend of abovementioned parameters was same, i.e., a highest increment was observed in AMF + DRGS followed by DRGS alone and AMF, respectively. However, their greatest enhancement was observed in the 2nd flowering and 2nd fruiting stages compared to the 1st flowering and 1st fruiting stages. Relative to the control, maximum content of proline, soluble sugar, and amino acid were observed under the AMF + DRGS treatment in the 2nd fruiting stage, which was 61.90, 39.90, and 32.73% higher than the control, respectively, followed by DRGS- and AMF-treated plants.

Figure 5D shows that the AMF, DRGS, and AMF + DRGS treatments enhanced the total phenolic content in eggplant leaves at all growth stages compared with the control. Total phenolic content increased from 1st flowering to 2nd fruiting stages. Total phenol was enhanced most in AMF + DRGS plants, in which it was 95.54% higher than the control. In AMF- and DRGS-treated plants, the corresponding increases were 38.65 and 60.80% (Figure 5D). Applying AMF alone or in combination with DRGS increased the leaf protein content (Figure 5E). The highest increase in leaf protein content was observed in the 2nd fruiting stage. Relative to the control, the protein content increased by 32.89, 37.31, and 46.17%, respectively, under the AMF, DRGS, and AMF + DRGS treatments.

Figure 5F shows the effect of AMF and DRGS upon the PAL activity at different growth stages of eggplant. PAL activity was evidently higher in AMF, DRGS, and AMF + DRGS plants than in the control. AMF-inoculated plants showed an increase of 19.29, 9.05, 14.05, and 18.73% in their PAL activity in the 1st flowering, 1st fruiting, 2nd flowering, and 2nd fruiting growth stages, respectively, compared with the control. The corresponding percentage increases under DRGS were 19.86, 28.27, 15.90, and 22.27%; however, the maximal increases of 24.00, 69.31, 26.02, and 34.61% were attained in AMF + DRGS-treated plants (Figure 5F).

### Arbuscular Mycorrhizal Fungi and Dry Raw Garlic Stalk Reduced Oxidative Stress Attributes and Enhanced Antioxidant Defense

**FIGURE 6:**
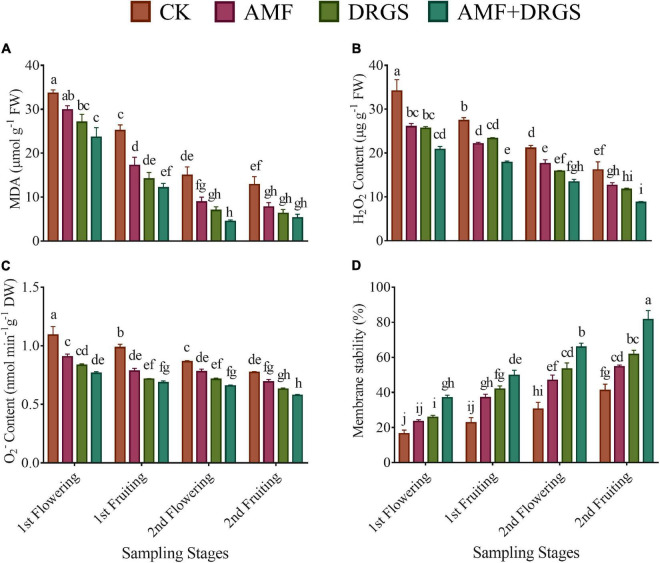
Effect of AMF inoculation and amendment with dry raw garlic stalk on (MDA) malondialdehyde **(A)**, hydrogen peroxide **(B)**, superoxide **(C)**, and membrane stability index **(D)** on eggplant leaves at 1st flowering, 1st fruiting, 2nd flowering, and 2nd fruiting stages. Data represent the mean of ±SE (*n* = 5). Different letters show significant differences within treatments. Tukey’s HSD test was employed to verify statistical significance at *p* < 0.05.

The AMF inoculation and DRGS amendment significantly reduced oxidative stress-related attributes compared with monocropped plants (i.e., the control) (Figures 6A–D). AMF- and DRGS-treated plants had reduced levels of H_2_O_2_ and O_2_^–^ production at all growth stages. The maximum reduction in H_2_O_2_ and O_2_^–^ content was observed in the 2^nd^ flowering and 2^nd^ fruiting stages compared to the 1^st^ flowering and 1^st^ fruiting stages (Figures 5B,C). Applying AMF and DRGS alone or in combination significantly reduced the production of both H_2_O_2_ and O_2_^–^. The most pronounced reduction in H_2_O_2_ occurred under the combined application of AMF + DRGS, which lowered it by 45.94% more than the control in the 2nd flowering and likewise 36.58% more in the 2nd fruiting stage (Figure 6B). Similarly, the maximum reduction in O_2_^–^ was observed under AMF + DRGS in the 2nd flowering (by 25.26%) and 2nd fruiting (24%) stages compared with the control, followed by the DRGS and AMF sole applications (Figure 6C). Furthermore, lipid peroxidation also increased in the control plants in the 2nd fruiting stage, yet it had declined in all treated plants (Figure 6A). Lipid peroxidation was reduced to the greatest extent in AMF + DRGS in the 2nd flowering (by 58.72%) and 2nd fruiting (70.03%) stages in comparison with control. Consequently, membrane stability increased in AMF- and DRGS-treated plants at all growth stages, but the highest increase (118.83%) happened under the AMF and DRGS combination in the 2nd fruiting stage (Figure 6D).

**FIGURE 7:**
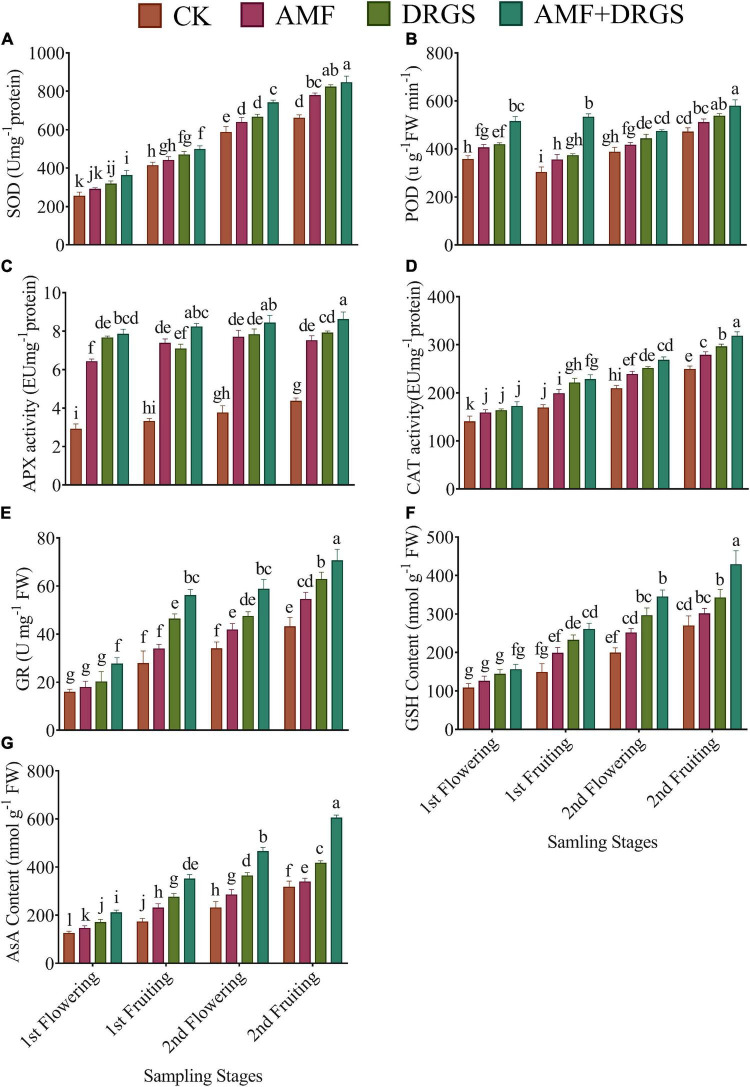
Effect of AMF inoculation and amendment with dry raw garlic stalk on superoxide dismutase (SOD) **(A)**, peroxidase (POD) **(B)**, ascorbate peroxidase (APX) **(C)**, catalase (CAT) **(D)**, glutathione reductase (GR) **(E)**, reduced glutathione (GSH) **(F)**, ascorbic acid (AsA) **(G)** on eggplant leaves at 1st flowering, 1st fruiting, 2nd flowering, and 2nd fruiting stages. Data represent the mean of ±SE (*n* = 5). Different letters show significant differences within treatments. Tukey’s HSD test was employed to verify statistical significance at *p* < 0.05.

Overall, the activities of antioxidant enzymes were significantly influenced by the application of AMF, DRGS, and their combined application at all growth stages. As seen in Figures 7A–G, compared with control, the AMF- and DRGS-treated plants featured increases in SOD, POD, APX, CAT, GR, GSH, and AsA activity. Yet, the maximum activity of these enzymes was reached in plants under the AMF + DRGS treatment. SOD activity was enhanced from the 1st flowering to 2nd fruiting stage but peaked in the 2nd fruiting stage in AMF + DRGS plants at 28.19% over the control, followed by DRGS (24.65%) and AMF (17.88%) (Figure 7A). POD activity was also significantly affected by the application of AMF and DRGS alone or in combination. POD activity increased at all growth stages. The highest increase in POD activity was observed in the 2nd fruiting stage *vis-à-vis* the other stages. Relative to the control, POD activity increased by 22.88% in AMF + DRGS plants, while it increased less so, by 14.20 and 8.48%, in DRGS- and AMF-treated plants, respectively. AMF and DRGS application alone or in combination induced a substantial rise in APX activity at all growth stages compared with the control (Figure 7C). APX activity increased from the 1st flowering to 2nd fruiting stages. APX activity peaked in the 2nd fruiting stage when it was 97.69% higher than the control, followed by the DRGS- and AMF-treated plants. Inoculation of AMF and DRGS amendment alone or in combination increased the CAT activity in comparison with the control (Figure 7D). CAT activity peaked in the 2nd fruiting stage under AMF + DRGS, at which time it was 52.04% higher than the control. It was increased by 26.45 and 34.20% in AMF- and DRGS-treated plants, respectively. Similarly, GR activity also increased in plants under AMF, DRGS, and AMF + DRGS treatments (Figure 7E). GR activity was greatest in the 2nd fruiting stage when it was 26.54, 34.20, and 52.04% higher than the control in AMF, GS, and AMF + DRGS plants, respectively.

Arbuscular Mycorrhizal Fungi or DRGS significantly increased the GSH and AsA activity in eggplants cultivated in CMC soil at all growth stages (Figures 7F,G). However, compared with the control, the highest increase in GSH (56.30%) and AsA (90.50%) was observed under the AMF + DRGS treatment in the 2nd fruiting stage.

### Effect of Arbuscular Mycorrhizal Fungi and Dry Raw Garlic Stalk on Hormones in Eggplant Leaves and Roots

**FIGURE 8:**
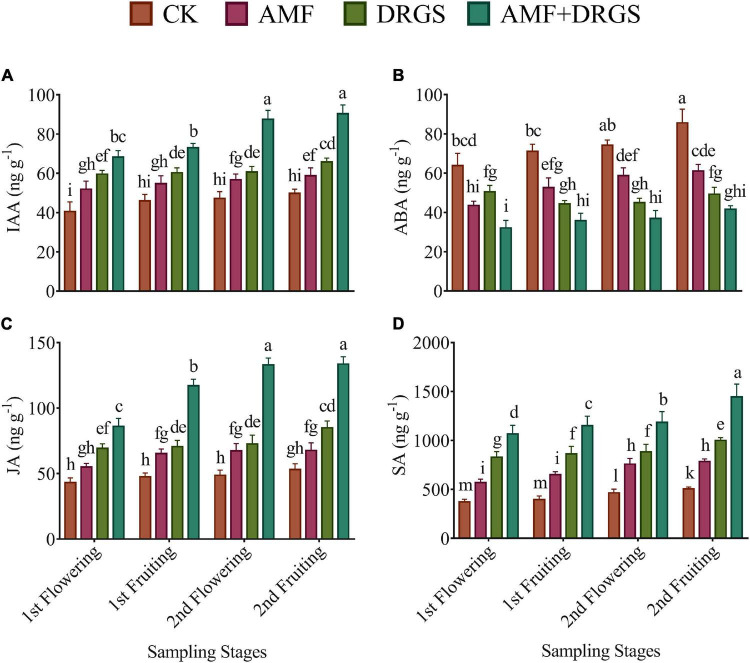
Effect of AMF inoculation and amendment with dry raw garlic stalk on phytohormones. Indoleacetic acid (IAA) **(A)**, abscisic acid (ABA) **(B)**, jasmonic acid (JA) **(C)**, salicylic acid (SA) **(D)** on eggplant leaves at 1st flowering, 1st fruiting, 2nd flowering, and 2nd fruiting stages. Data represent the mean of ±SE (*n* = 5). Different letters show significant differences within treatments. Tukey’s HSD test was employed to verify statistical significance at *p* < 0.05.

Incorporating AMF, DRGS, and AMF + DRGS in continuous cropping soil significantly increased the level of IAA, JA, and SA in eggplant leaves from 1st flowering through 2nd fruiting stages (Figures 8A–D). However, the content of ABA was always higher in control plants. The greatest levels of these hormones were observed in the 2nd fruiting stage in the AMF + DRGS plants. Compared with the control, the level of IAA, SA, and JA in AMF + DRGS plants increased by 81.51, 185.29, and 151.61%, respectively; under AMF, they increased by 28.02, 53.54, and 27.39%, respectively; while, under DRGS, they increased by 68.37, 123.03, and 60.47%, respectively (Figure 8B). The most pronounced decline was observed in the 2nd fruiting stage in AMF + DRGS-treated plants at 51.36% relative to the control. Inoculation with AMF decreased ABA by 48.42%, and, under the DRGS treatment, it was reduced by 28.63%.

Table 2 reports the effects of AMF and DRGS applications alone or in combination upon the hormonal levels in eggplant roots. The levels of IAA, SA, and JA hormones increased in plants under the AMF, DRGS, and combined application of AMF + DRGS when compared to control; however, ABA in roots showed a trend of decreasing in all treated plants. The maximum increase in IAA, SA, and JA was observed in the AMF + DRGS plants, being respectively 102.84, 86.63, and 91.08% greater than the control plants. Compared with the control, IAA, SA, and JA levels were increased by 32.34, 41.36, and 39.93%, respectively, by AMF alone, while, under DRGS, corresponding percentage increases were 62.13, 70.60, and 71.37%.

**TABLE 2:** Effect of AMF inoculation and DRGS treatment on mineral nutrients in eggplant leaves and phytohormones in roots.

Treatment	N (mg g^–1^DW)	P (mg g^–1^DW)	K (mg g^–1^DW)	IAA (ng g^–1^)	ABA (Ng g^–1^)	JA (ng g^–1^)	SA (ng g^–1^)
CK	12.15 ± 0.17d	14.15 ± 0.83d	9.73 ± 0.41d	11.65 ± 0.99d	21.38 ± 1.27a	34.01 ± 2.36d	276.67 ± 9.02d
AMF	17.13 ± 0.56c	18.75 ± 0.13c	11.21 ± 0.57c	15.41 ± 1.07c	18.28 ± 0.54b	47.59 ± 1.85c	391.12 ± 8.38c
GS	19.29 ± 0.54b	22.21 ± 0.46b	13.21 ± 0.45bc	18.83 ± 1.16b	13.81 ± 1.06c	58.29 ± 2.54b	472.00 ± 7.25b
AMF + GS	21.87 ± 0.89a	25.12 ± 0.20a	15.77 ± 0.15a	23.59 ± 1.33a	8.19 ± 0.27d	65.00 ± 1.02a	516.38 ± 8.46a

*Data represent the mean of ±S.E. (n = 5). Different letters show significant differences within treatments. Tukey’s HSD test was employed to verify statistical significance at p < 0.05.*

### Effect of Arbuscular Mycorrhizal Fungi and Dry Raw Garlic Stalk on Eggplant Leaves’ Mineral Nutrients

Continuous monocropping plants significantly reduced the mineral nutrients in eggplant leaves compared to AMF, DRGS, and the combined application of AMF + DRGS. The highest N, P, and K content were recorded in AMF + DRGS-treated plants, being 80, 77.52, and 62.07% higher than the control (Table 2).

### Arbuscular Mycorrhizal Fungi and Dry Raw Garlic Stalk Reduced *Fusarium* Wilt and Increased Eggplant’s Yield

Applying AMF, DRGS, or AMF + DRGS had a significant effect on the control of *Fusarium* wilt disease. The disease incidence declined under AMF, DRGS, and AMF + DRGS treatments by 44, 55, and 77%, respectively, *vis-à-vis* the control (Table 1). Relative to the control, eggplant yield was enhanced by 31.08, 40.59, and 79.40% in response to AMF, DRGS, and AMF + DRGS, respectively (Table 1).

### Correlation Between Physiological, Biochemical Traits, and Eggplant Yield

**FIGURE 9:**
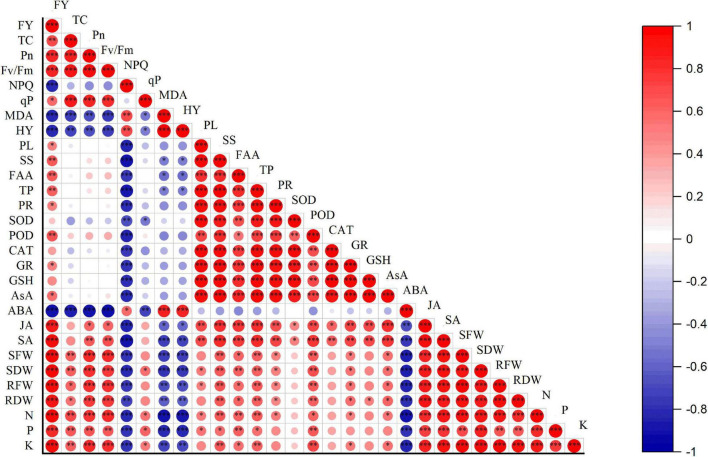
Correlation between growth, physiological, biochemical traits, and eggplant yield. Pearson correlation between eggplant growth, physiological, biochemical, and yield-related parameters. The red color represents a positive correlation, while the blue color means a negative correlation. The lighter colors and small circles indicate the lower values of the correlation coefficient, whereas darker colors and bigger circles show a high positive correlation. FY, fruit yield; TC, total chlorophyll; Pn, photosynthesis; *F*_v_/*F*_m_, maximal photochemical efficiency; NPQ, non-photochemical quenching coefficient; qP, photochemical quenching; MDA, malonaldehyde; H_2_O_2_, hydrogen peroxide; PL, proline; SS, soluble sugar; FAA, free amino acid; TP, total phenols; SOD, POD, CAT, GR, GSH, AsA, superoxide dismutase, peroxidase, catalase, glutathione reductase, reduced glutathione, ascorbic acid, respectively. ABA, JA, SA, abscisic acid, jasmonic acid, salicylic acid, respectively. SFW, shoot fresh weight; SDW, shoot dry weight; RFW, root fresh weight; RDW, root dry weight; N, P, K, mineral nutrients. The stars indicate a significant correlation at (*) 0.05, (^**^) 0.01, (^***^), and 0.001 levels of significance.

A Pearson correlation was performed among different physiological, biochemical, and yield of eggplant (Figure 9). The analysis exhibited a highly significant positive correlation among eggplant yield TC, Pn, *F*_V_/*F*_m_, antioxidant enzymes, JA, SA, SFW, SDW, RFW, RDW, and leaf mineral nutrients. However, a highly negative correlation was observed between NPQ, MDA, HY, ABA, and eggplant yield. TC has a significantly positive correlation with Pn, *F*_v_/*F*_m_, qp, phytohormones, shot fresh weight and dry weight, and leaves’ mineral nutrients. A highly negative correlation was observed between NPQ, MDA, HY, and ABA. NPQ has a positive correlation with MDA, HY, and ABA. However, NPQ showed a negative correlation between different physiological and biochemical indexes. MDA has a positive correlation between HY and ABA. A highly negative correlation was observed between phytohormones, leaf mineral nutrients, shoot fresh and dry weight, root fresh and dry weight, and MDA. The similar trend was observed between HY and other physiological and biochemical parameters. Osmolytes have a positive correlation with antioxidant enzymes, JA, SA, shoot fresh and dry weight, root fresh and dry weight, and leaf mineral nutrients. Plant leaf antioxidants positively correlate with JA, SA, SFW, SDW, RFW, RDW, and plant leaf mineral nutrients, and a weak negative correlation was found between ABA and antioxidant enzymes. Phytohormones such as JA and SA positively correlate with physiological and biochemical parameters. ABA was positively correlated with HY, MDA, and NPQ, and a negative correlation was found between ABA and physiological and biochemical indexes. RFW, RDW, leaf mineral nutrients, and physiological, biochemical, and phytohormone (SA and JA) indexes were all linked to these indexes. However, a negative correlation was found between the aforementioned parameters and HY, MDA, NPQ, and ABA. The results revealed that eggplant fruit yield strongly depends on morphological and physiological characteristics.

## Discussion

The mismanagement of crop fields has led to considerable decline in the soil quality of agricultural fields. Reduced soil health influences the growth and productivity of crops by restricting key cellular processes in plants. Monocropping is one of the agricultural practices that adversely affect the growth and productivity of many crops in major parts of the globe, including China. Against this backdrop, the beneficial effects of a DRGS amendment and an AMF inoculation for ameliorating the negative impact of CMC on eggplant were investigated in this study, and how they influenced this plant’s major tolerance mechanisms was studied. The results demonstrated that monocropping severely impacted multiple growth attributes, including plant height, stem diameter, plant fresh and dry weight, root fresh and dry weight, root activity, and root length. However, applying DRGS and AMF to eggplant, either alone or in combination, significantly mitigated its growth decline caused by CMC. These observations are supported by our previous findings of the growth-promoting effects of DRGS and AMF on cucumber and eggplant grown in continuous cropping soil (Ali et al., 2019a; Ghani et al., 2019a). Similarly, other work has shown that AMF inoculation eliminates the negative effect of CMC and promotes crop growth by stimulating changes in morphological and biochemical attributes (Ortas, 2012; Li et al., 2019; Liu et al., 2020). Plants inoculated with AMF undergo an increase in their roots-related parameters in continuous cropping soil (Ortas, 2012; Wang et al., 2021). Previous studies have explored the relationship between root elongation and root activity (Bi et al., 2018; Moore et al., 2020), which contribute to greater nutrient acquisition and water uptake *via* mycorrhizal hyphae (Jackson et al., 2019; El Amerany et al., 2020). In our study, plants inoculated with AMF alone or in combination with DRGS had improved root-related parameters. Sole application of DRGS, AMF, or their combination, increased root length along with root activity in continuous cropping soil (Ghani et al., 2019a; Liu et al., 2020).

The AMF colonization is an essential index for determining the amount of infection in host plants. In our study, colonization percentage was lower in plants treated with AMF only than those treated with combined AMF and DRGS. This result is consistent with findings of Ali et al. (2019a) and Li et al. (2019), which suggests that the percentage of AMF colonization in CMC soil is reduced because host plant roots and traits of colonized AM fungi are sensitive to the exogenous phosphorus (P) supply. Hence, AMF root colonization may be decoupled from the soil due to the limited or excessive supply of P. Additionally, external hyphae of AMF need 4–7 times higher nitrogen (N) content than do plant shoots and 10 times what roots require. Therefore, AMF need considerable nitrogen for their growth and structural development (Hodge and Fitter, 2010). In contrast, combining AMF with an organic substrate input increases the percentage of roots colonized; the organic substrate provides additional carbon and other important nutrients, potentially suppressing some soil-borne pathogens, thereby accelerating AMF colonization (Ali et al., 2019a; Jackson et al., 2019).

The DRGS and AMF treatments in our study increased both plant growth and aboveground biomass accumulation, which can be attributed to a higher absorption of mineral ions, such as N, P, and potassium (K). Monocropped plants incur significant declines in mineral nutrients under non-inoculated conditions, yet the plants inoculated with AMF had greater levels of N, P, and K (Ortas, 2012; Li et al., 2019; Wang et al., 2021). Mineral nutrients such as N and K play an important role in plant development by regulating enzyme activity, membrane permeability, and osmolyte accumulation, and contributing to their ability to counter different biotic and abiotic stresses (Begum et al., 2020; Ahanger et al., 2021).

Photosynthesis is pivotal to maintaining and enhancing plant growth and, as a result, enhances the capacity of tolerance in plants to deal with various environmental stresses (Ahanger et al., 2021). The mechanism of chlorophyll fluorescence has been widely used in plant physiology and is crucial for understanding the mechanism underpinning photosynthetic responses to environmental changes. Our experiment’s results demonstrate that applying AMF and DRGS alone or in combination significantly enhanced total chlorophyll, Pn, E, Ci, and PSII activity. Furthermore, different biotic and abiotic stresses cause pigments’ breakdown by increasing chlorophyllase activity and decreasing the activity of enzymes involved in chlorophyll production, thereby affecting the chlorophyll fluorescence as well as net photosynthesis (Ashraf and Harris, 2013). Our findings agree with several previous studies (Ali et al., 2019a; Gómez-Bellot et al., 2021), demonstrating that organic substrate and AMF enhanced Pn and stomatal conductance in monocropped soil.

The joint application of AMF and DRGS significantly decreased the oxidative damage under monocropping caused by environmental stresses. By increasing ROS production, differing environmental stresses can weaken plant vigor and fitness (Wang et al., 2015). Levels of ROS, such as H_2_O_2_ and O_2_^–^, were considerably lower in AMF- and DRGS-treated plants, resulting in lower their lipid peroxidation and increased membrane stability. Overproduction of ROS levels causes oxidation, damages membrane proteins and lipids, which leads to poor plant growth (Ahanger et al., 2021; Ghani et al., 2022). Current research indicates that biomass production and growth maintenance in AMF-inoculated plants are linked with decreased O_2_^–^, H_2_O_2_, and lipid peroxidation under stressful conditions *vis-à-vis* non-AMF inoculated plants. AMF symbioses protect plants by interfering with the pathway of ROS generation, thereby maintaining membrane stability and stabilizing the proteins and enzymes. Less oxidative damage may be attributed to a bolstered antioxidant system in AMF- and DRGS-treated plants that enables the quick removal of excess ROS. Superoxide is scavenged by SOD, whereas H_2_O_2_ is neutralized by either POD at the membranes or CAT in the cytosol, or APX and GR in the mitochondria and chloroplast through the AsA-GSH cycle (Begum et al., 2020; Ahanger et al., 2021). Previous studies suggest that AMF inoculation causes an increase in the functioning of the antioxidant system (Begum et al., 2020; Wang et al., 2021). It has been shown that AMF inoculation improves the antioxidant mechanism, resulting in enhanced photosynthesis in eggplant and tomato cultivated in continuously cropped soil (Ortas, 2012; Wang et al., 2021). Hence the photoprotection observed in our study here may be attributed to an activated antioxidant system in DRGS- and AMF-treated plants, which could be explained by (1) rapid removal of ROS, and (2) maintenance of redox equilibrium *via* enhanced GSH and AsA production. Furthermore, applying AMF and DRGS together augmented the accumulation of POD, SOD, APX, GR, and CAT, and the higher level of GSH and ASA had more effectively mitigated oxidative damage compared with the control. AMF inoculation strengthens the antioxidant system by maintaining the redox equilibrium, resulting in the protection of vital metabolic processes such as photosynthesis (Begum et al., 2020). GSH plays an essential role in protecting membrane stability by keeping the α-tocopherol and zeaxanthin in a reduced state (Ahanger et al., 2021).

Glutathione preserves the thiol groups of proteins under stress, limiting their oxidative damage, and it is thought to be involved in regulating phytohormones (Begum et al., 2020; Ahanger et al., 2021). The integration in plants of the antioxidant system with hormonal regulation promotes their tolerance to various environmental stresses (Begum et al., 2020). These phytohormones, namely IAA, ABA, SA, and JA, are essential signaling molecules, and the upregulation of their synthesis during stressful conditions improves plant growth and yield (Ahanger et al., 2021; Ali M. et al., 2021; Wang et al., 2021). In our study, applying AMF or DRGS alone or combined significantly increased the IAA, SA, and JA concentrations in both the leaves and roots of eggplant; however, its ABA concentration was significantly increased in control plants compared with the AMF and DRGS treatments. Low level of ABA stimulates root growth vis- -vis high concentration diminished root growth (Xu et al., 2013). Similar results were observed by Wang et al. (2021), where the IAA level increased after AMF inoculation and ABA decreased in tomato roots and leaves in monocropped soil conditions. However, Abd_Allah et al. (2015) found that AMF inoculation under water deficit conditions reduced the levels of growth-promoting hormones (IAA and CTK) and substantially increased the ABA level; they also observed that AMF could ameliorate the hormonal imbalance caused by drought stress. Our findings show that, rather than hormonal changes, AMF may have alleviated monocropping-induced stress by increasing the level of IAA, SA, JA, and reducing that of ABA.

Recent findings have revealed that inoculation with AMF can increase the IAA in leaves and roots in monocropped soil (Wang et al., 2021). Phytohormones such as JA and SA are primarily recognized for their functions in regulating plant stress responses to pathogen attack. Upon pathogen detection, infected plant cells produce phytohormone-signaling molecules that activate the defense system in neighboring cells and inhibit the pathogen’s spread (Kazan and Lyons, 2014). In our study, the levels of JA and SA both increased under the AMF and DRGS treatments, and their combined application. Furthermore, the incidence of *Fusarium* wilt was also reduced in AMF- and DRGS-treated eggplant. Our results are consistent with previous study of Ali M. et al. (2021) and Mishev et al. (2021) where AMF and garlic-derived allelochemicals regulated the phytohormones-signaling pathway and suppressed disease-causing pathogens in eggplant and tobacco. Furthermore, they also play an important role in regulating certain plant physiological responses, such as the antioxidant system, amino acid buildup, and soluble sugar levels (Ali M. et al., 2021). In our study, the concentrations of JA and SA were higher in AMF- and DRGS-treated eggplant than the control, which agrees with other work that reported inoculation with AMF increased the JA and SA concentrations in tomato plants cultivated in monocropped soil (Wang et al., 2021). It is well documented that the buildup of compatible solutes, including proline, amino acid, and sugar, are fundamental strategies for the survival and defense of plants under stressful conditions. The AMF inoculation and DRGS amendment significantly improved osmolytes, such as proline, free amino acids, and sugar contents, in eggplant leaves compared with control. AMF inoculation was found to enhance osmolyte accumulation in tomato seedlings (Mona et al., 2017). Thus, plants are likely to downregulate the catabolism of osmolytes to exploit their positive effects for stress tolerance.

Eggplants grown in continuous cropping soil show diminished quality induced by the accumulation of soil-borne diseases that result in a decline of their yield. *Fusarium* wilt is a fungal disease caused by the soil-borne fungus *Fusarium oxysporum*, capable of severely impairing eggplant production (Chakraborty et al., 2009). However, the use of fungicides may negatively impact the local environment. By contrast, inoculation with AMF and amendment of soil with organic residues is an environmentally friendly way to reduce the severity of this disease. In our study, the combined application of AMF and DRGS significantly reduced disease severity and increased the eggplant yield. The observed reduction in the severity of *Fusarium* incidence is because the strength from the dual application of AMF and DRGS generated a more efficient initiation of rooting structures and increased root activity, thereby positively impacting the yield of eggplant. The pragmatic bio-efficiency of this research might explain why garlic or garlic-derived components—flavonoids, phenolic, sulfur-containing compounds, antioxidant enzymes, and carbohydrates—have been recognized as antibacterial agents as well as biostimulants. Furthermore, applying AMF and DRGS increased the total phenol compared to monocropping with such treatments. Phenols can act as electron donors for peroxidase (PODs), which protect the cell wall and membrane components against ROS-mediated damage, and they can also serve as signals for the establishment of AMF symbiosis (Mandal et al., 2010). As a result, AMF and DRGS mediated their greater accumulation, contributing to better crop growth by fostering and sustaining the beneficial symbiosis with AMF.

## Conclusion

Within the context of the transition of agricultural practices toward sustainable vegetable cultivation, using dry garlic stalk waste (DRGS) and arbuscular mycorrhizal fungi (AMF) is a viable solution to the environmental degradation resulting from monocropping. The present research findings demonstrate that AMF and DRGS applications significantly mitigated continuous cropping-related growth decline obstacles by improving the photosynthesis, chlorophyll content, root activity, mineral uptake, and plant biomass of eggplant. Furthermore, positive effects were attained by modulation of the plant antioxidant defense system, osmolytes, phytohormones, and secondary metabolites. In addition, the beneficial effects were starkly evinced by the lower ROS accumulation translating into reduced lipid peroxidation and increased structural and functional stability of cell wall membranes.

## Data Availability Statement

The original contributions presented in the study are included in the article/Supplementary Material, further inquiries can be directed to the corresponding author/s.

## Author Contributions

MG: conceptualization, data curation, formal analysis, investigation, methodology, software, writing – original draft preparation, and writing – review and editing. AA: validation, investigation, and writing – review and editing. MJA: conceptualization, writing, and editing. MuA: data curation and validation. BA: investigation and data curation. ZC: conceptualization, funding acquisition, investigation, methodology, project administration, and supervision. All authors contributed to the manuscript.

## Conflict of Interest

The authors declare that the research was conducted in the absence of any commercial or financial relationships that could be construed as a potential conflict of interest.

## Publisher’s Note

All claims expressed in this article are solely those of the authors and do not necessarily represent those of their affiliated organizations, or those of the publisher, the editors and the reviewers. Any product that may be evaluated in this article, or claim that may be made by its manufacturer, is not guaranteed or endorsed by the publisher.
