# Supplementary figures and images for: Arbuscular Mycorrhizal Fungi and Dry Raw Garlic Stalk Amendment Alleviate Continuous Monocropping Growth and Photosynthetic Declines in Eggplant by Bolstering Its Antioxidant System and Accumulation of Osmolytes and Secondary Metabolites
Source: Front Plant Sci. 2022 Mar 31;13:849521. doi: 10.3389/fpls.2022.849521 (PMC9008779; doi:10.3389/fpls.2022.849521)

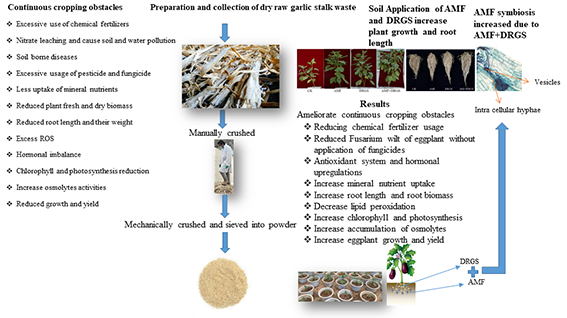

Supplement: Supplementary file 1 [file Image_1.TIFF]
