# Supplementary material for: Arbuscular Mycorrhizal Fungi and Dry Raw Garlic Stalk Amendment Alleviate Continuous Monocropping Growth and Photosynthetic Declines in Eggplant by Bolstering Its Antioxidant System and Accumulation of Osmolytes and Secondary Metabolites
Source: Front Plant Sci. 2022 Mar 31;13:849521. doi: 10.3389/fpls.2022.849521 (PMC9008779; doi:10.3389/fpls.2022.849521)
Supplement: Supplementary file 2 [file Table_1.DOCX]

**Supplementary Table**

**Table S1** Basic characteristics of DRGS and soil before experiment

| Parameters | Soil | Garlic stalk |
| --- | --- | --- |
| Soil Type | Brown -loamy-alkaline-Orthic Anthrosol |  |
| pH | 7.64 ± 0.50 | 7.45 ± 0.60 |
| EC (µS • cm^−1^) | 383 ± 6.00 | 620 ± 10.50 |
| Organic Carbon (g.kg^-1^) | 15.13 ± 0.90 | 370.08 ± 8.50 |
| Total Nitrogen (g.kg^-1^) | 1.14 ± 0.06 | 8.07 ± 0.50 |
| C:N | 13.42 ± 0.80 | 45.85 ± 5.50 |
| Organic Matter (g.kg^-1^) | 12.97 ± 0.50 | 639.25 ± 12.50 |
| Total Phosphorus (g.kg^-1^) | 0.91 ± 0.030 | 12.87 ± 1.50 |
| Total Potassium (g.kg-1) | 6.87 ± 0.50 | 10.19 ± 0.05 |
| Available N (mg.kg^-1^) | 60.3 ± 3.50 | - |
| Available P (mg.kg^-1^) | 55.01 ± 5.10 | - |
| Available K (mg.kg^-1^) | 189 ± 4.80 | - |
| Soil Planting History | Eggplant continuously planted for five years |  |
|  |  |  |
